# State-Specific Prevalence of Tobacco Product Use Among US Women, Tobacco Use Supplement to the Current Population Survey, 2018–2019

**DOI:** 10.5888/pcd18.200547

**Published:** 2021-04-15

**Authors:** Teresa W. Wang, Kimp Walton, Carolyn Reyes-Guzman, Karen A. Cullen, Ahmed Jamal

**Affiliations:** 1 Office on Smoking and Health, National Center for Chronic Disease Prevention and Health Promotion, Centers for Disease Control and Prevention, Atlanta, Georgia; 2 Tobacco Control Research Branch, Division of Cancer Control and Population Sciences, National Cancer Institute, Bethesda, Maryland; 3 Center for Tobacco Products, Food and Drug Administration, Silver Spring, Maryland

## Abstract

In this study, we assessed tobacco product use among US women aged 18 years or older using data from the 2018–2019 Tobacco Use Supplement to the Current Population Survey. State-specific current use of any tobacco product (cigarettes, e-cigarettes, cigars, regular pipes, water pipes or hookah, and smokeless tobacco) ranged from 6.6% (California) to 23.1% (West Virginia); current use of 2 or more tobacco products ranged from 0.6% (New York) to 3.0% (Oklahoma). Current tobacco product use among US women differed significantly by age, education, race/ethnicity, household income, marital status, disability status, and US region. Comprehensive tobacco control strategies, including targeted interventions, can reduce tobacco use among all women.

**Summary**

**What is already known on this topic?**

Tobacco product use among women is associated with substantial morbidity and mortality, including sex-specific health consequences.

**What is added by this report?**

We found that current use of any tobacco product among women significantly differed by age, race/ethnicity, education level, annual household income, marital status, disability status, and US region.

**What are the implications for public health practice?**

By state, at least 1 in 15 women currently used some form of tobacco product. Comprehensive tobacco control strategies, including targeted interventions among subgroups with higher rates of use, can prevent and reduce tobacco product use among all women.

## Objective

Tobacco product use among women is associated with substantial morbidity and mortality, including sex-specific health consequences; each year, approximately 202,000 US women die of smoking-related diseases (1–3). Although overall US smoking prevalence is consistently lower among women compared with men, estimates have not declined commensurately among women over time (2). As the tobacco product landscape continues to diversify, data characterizing women’s tobacco product use behaviors can help inform public health policy, planning, and practice. We used data from the 2018–2019 Tobacco Use Supplement to the Current Population Survey (TUS-CPS) to estimate state-specific prevalence and correlates of current tobacco product use among US women.

## Methods

Data came from the 2018–2019 TUS-CPS, a cross-sectional, household-based survey of noninstitutionalized US adults aged 18 years or older in the 50 US states and the District of Columbia (DC) (4,5). Using probability-based multi-stage sampling, respondents in the 2018–2019 TUS-CPS were interviewed once either in July 2018, January 2019, or May 2019. Excluding proxy respondents, 137,471 self-respondents completed the interview in-home or by telephone (self-response rate, 57.6%).

We limited our analysis to women (N = 74,983) and applied self-response survey weights. Any tobacco product use was defined as use of at least 1 of the following products: cigarettes, e-cigarettes, cigars, water pipes or hookah, smokeless tobacco (including moist snuff, dip, spit, chew tobacco, snus, or dissolvable tobacco), and regular pipes. Current cigarette smokers were defined as adults who smoked at least 100 cigarettes during their lifetime and smoked “every day” or “some days” at the time of survey administration. For all other products, current use was defined as use one or more times during their lifetime and “every day” or “some days” at the time of survey administration.

State-specific prevalence estimates and 95% CIs were generated for all 50 states and DC for current use of any tobacco product, 2 or more products, and each product. Model-adjusted prevalence ratios (aPRs) with predicted marginals accounting for age, race/ethnicity, education, annual household income, marital status, disability status, and US Census region were calculated for any current tobacco product use, cigarette smoking, and e-cigarette use. Unstable estimates with relative standard errors higher than 30% were suppressed. Analyses were performed using SAS-callable SUDAAN version 11.0.1 (RTI). Standard errors were estimated using the Fay-adjusted Balanced Repeated Replication method. Additional details are available at http://www.cancermeetings.org/TUSCPSWebinar/documents/tuscps_Webinar_Liu.pdf.

## Results

State-specific prevalence of any current tobacco product use among women ranged from 6.6% (California) to 23.1% (West Virginia) (Table 1). Current use of 2 or more tobacco products ranged from 0.6% (New York; representing 6.4% of any tobacco product users) to 3.0% (Oklahoma; 15.3% of any tobacco product users). Cigarettes were the most commonly used product among women in all states and DC (range, 5.5% in California to 21.3% in West Virginia), followed by e-cigarettes (range, 1.1% in California and New York to 5.1% in Oklahoma). Current cigar smoking ranged from 0.3% (California) to 1.5% (Oklahoma, Louisiana). Current hookah smoking ranged from 0.4% (California) to 1.7% (DC). All state-specific estimates for smokeless tobacco use and regular pipe smoking either did not meet stable reporting thresholds or had zero respondents report use of the corresponding product.

**Table 1:** Prevalence of Current Use of Tobacco Products among Women Aged ≥18 Years, by State, Tobacco Use Supplement to the Current Population Survey, United States, 2018–2019

State	Any Tobacco Product^a^^,^ b	≥2 Tobacco Products^c^	Cigarettes^d^	E-cigarettes^e^
	% (95% CI)	% (95% CI)	% (95% CI)	% (95% CI)
National	11.6 (11.3–11.9)	1.1 (1.0–1.2)	10.0 (9.7–10.3)	1.8 (1.7–1.9)
Alabama	15.0 (12.8–17.5)	1.6 (0.9–2.8)	13.5 (11.6–15.7)	2.0 (1.2–3.3)
Alaska	13.9 (11.4–16.9)	–f	12.7 (10.5–15.2)	–f
Arizona	10.0 (8.3–12.1)	1.6 (1.0–2.6)	8.3 (6.8–10.1)	2.1 (1.3–3.6)
Arkansas	15.1 (12.8–17.8)	2.0 (1.1–3.5)	13.7 (11.4–16.3)	2.2 (1.4–3.5)
California	6.6 (5.9–7.3)	0.7 (0.5–1.0)	5.5 (4.9–6.1)	1.1 (0.8–1.5)
Colorado	8.8 (6.9–11.3)	–f	7.3 (5.4–9.7)	1.9 (1.1–3.3)
Connecticut	9.5 (7.1–12.6)	–f	7.5 (5.4–10.5)	–f
Delaware	9.5 (7.3–12.5)	–f	8.5 (6.5–11.1)	–f
District of Columbia	11.0 (9.4–12.9)	–f	9.1 (7.6–10.8)	–f
Florida	10.4 (9.2–11.7)	1.2 (0.8–1.7)	8.9 (7.8–10.0)	1.5 (1.0–2.1)
Georgia	12.7 (11.3–14.2)	0.9 (0.5–1.5)	10.7 (9.5–12.1)	1.3 (0.9–1.9)
Hawaii	8.8 (6.7–11.6)	–f	8.3 (6.2–11.0)	–f
Idaho	11.3 (9.3–13.7)	–f	9.9 (7.9–12.2)	2.0 (1.4–3.0)
Illinois	11.9 (10.1–14.0)	1.1 (0.7–1.8)	10.6 (8.8–12.6)	2.0 (1.4–2.8)
Indiana	14.9 (12.8–17.4)	1.3 (0.8–2.1)	12.9 (10.7–15.6)	2.6 (1.8–3.7)
Iowa	16.3 (13.1–20.0)	1.9 (1.1–3.2)	14.8 (11.9–18.3)	2.8 (1.9–4.1)
Kansas	14.7 (11.9–17.9)	–f	12.5 (10.0–15.4)	–f
Kentucky	18.7 (16.4–21.2)	1.7 (1.0–2.7)	16.1 (14.0–18.5)	3.1 (2.0–4.8)
Louisiana	14.4 (12.6–16.4)	1.2 (0.8–2.0)	13.4 (11.7–15.3)	–f
Maine	15.8 (13.4–18.6)	2.6 (1.6–4.3)	14.2 (12.1–16.7)	2.8 (1.6–4.9)
Maryland	11.5 (9.3–14.1)	–f	8.7 (7.0–10.9)	2.1 (1.3–3.6)
Massachusetts	9.6 (7.9–11.6)	0.9 (0.5–1.5)	8.4 (6.9–10.3)	1.7 (1.1–2.8)
Michigan	14.2 (12.3–16.3)	–f	12.7 (11.1–14.6)	1.2 (0.6–2.1)
Minnesota	11.9 (9.5–14.7)	–f	10.5 (8.4–13.0)	1.6 (1.0–2.7)
Mississippi	15.4 (13.8–17.1)	1.5 (0.8–2.5)	14.1 (12.7–15.6)	–f
Missouri	16.4 (14.1–19.0)	1.9 (1.1–3.3)	13.7 (11.5–16.3)	3.1 (2.1–4.6)
Montana	16.2 (14.2–18.5)	–f	14.4 (12.6–16.5)	2.0 (1.2–3.2)
Nebraska	15.7 (13.4–18.5)	1.4 (0.8–2.5)	13.8 (11.3–16.7)	2.2 (1.4–3.5)
Nevada	12.1 (10.2–14.3)	1.2 (0.7–2.1)	10.7 (8.8–13.0)	–f
New Hampshire	14.3 (11.7–17.5)	–f	11.9 (9.8–14.4)	–f
New Jersey	9.2 (7.5–11.2)	–f	6.9 (5.6–8.5)	2.4 (1.5–3.7)
New Mexico	11.2 (9.3–13.5)	–f	10.0 (8.1–12.3)	1.3 (0.8–2.2)
New York	9.9 (8.8–11.1)	0.6 (0.4–1.1)	8.5 (7.6–9.6)	1.1 (0.7–1.6)
North Carolina	13.5 (11.5–15.7)	1.4 (1.0–1.9)	11.7 (9.9–13.7)	2.0 (1.6–2.6)
North Dakota	15.9 (13.0–19.3)	–f	14.4 (11.9–17.5)	–f
Ohio	16.9 (15.2–18.8)	1.8 (1.2–2.5)	14.7 (12.9–16.5)	2.7 (2.0–3.6)
Oklahoma	19.7 (17.0–22.7)	3.0 (2.0–4.4)	15.3 (12.8–18.2)	5.1 (3.7–7.0)
Oregon	13.2 (10.9–15.9)	1.5 (0.9–2.5)	10.8 (8.8–13.2)	2.7 (1.7–4.2)
Pennsylvania	15.3 (13.8–17.1)	1.7 (1.1–2.6)	13.9 (12.3–15.6)	2.2 (1.6–3.0)
Rhode Island	12.2 (9.6–15.4)	–f	10.1 (7.9–12.9)	–f
South Carolina	11.6 (9.5–14.0)	–f	10.4 (8.4–12.9)	1.6 (1.0–2.7)
South Dakota	16.0 (13.8–18.5)	–f	13.8 (11.4–16.6)	3.2 (1.8–5.4)
Tennessee	15.2 (13.4–17.3)	0.7 (0.4–1.2)	13.8 (12.1–15.6)	1.4 (0.9–2.2)
Texas	8.4 (7.5–9.4)	0.8 (0.6–1.1)	6.7 (5.9–7.6)	1.8 (1.4–2.3)
Utah	7.3 (5.0–10.7)	–f	5.8 (3.8–8.6)	1.7 (1.0–3.0)
Vermont	11.5 (9.4–14.1)	–f	10.0 (8.0–12.3)	1.7 (1.0–3.0)
Virginia	9.9 (7.8–12.5)	1.2 (0.7–2.1)	8.5 (6.6–10.9)	1.8 (1.2–2.8)
Washington	10.7 (9.0–12.6)	1.0 (0.5–1.7)	9.3 (7.8–11.1)	1.6 (1.0–2.6)
West Virginia	23.1 (19.2–27.6)	2.4 (1.6–3.4)	21.3 (17.7–25.3)	3.2 (2.2–4.6)
Wisconsin	14.8 (12.8–17.1)	–f	13.1 (11.1–15.4)	2.5 (1.5–4.1)
Wyoming	16.0 (13.1–19.3)	1.2 (0.7–2.2)	12.5 (10.2–15.2)	3.4 (2.1–5.4)

Abbreviation: e-cigarette, electronic cigarette.

a Women who reported ever use of at least 1 of the 6 tobacco products assessed (cigarettes, e-cigarettes, cigars, water pipe or hookah, smokeless tobacco [moist snuff, dip, spit, chew tobacco, or snus], and regular pipe) and reported using the respective product “every day” or “some days” at the time of the survey. For cigarettes only, users were defined as women who had smoked ≥100 cigarettes during their lifetime and now smoked cigarettes either “every day” or “some days” at the time of survey administration.

b State-specific columns are not presented for current use of 4 tobacco product types: cigars (overall weighted, 0.6% [95% CI, 0.5%–0.7%]); smokeless tobacco (overall weighted, 0.1% [95% CI, 0.1%–0.1%]); water pipe or hookah (overall weighted, 0.3% [95% CI, 0.3%–0.4%]); and regular pipe (overall weighted, 0.05 [95% CI, 0.03%–0.07%]). Prevalence of current cigar smoking was 0.3% (95% CI, 0.2%–0.5%) in California, 0.8% (95% CI, 0.5%–1.2%) in Florida, 0.8% (95% CI, 0.5%–1.4%) in Georgia, 1.5% (95% CI, 0.9%–2.3%) in Louisiana, 1.5% (95% CI, 0.8%–2.6%) in Oklahoma, 0.5% (95% CI, 0.3%–0.8%) in Texas, and 0.5% (95% CI, 0.3%–0.9%) in West Virginia. Prevalence of current hookah smoking was 0.4% (95% CI, 0.3%–0.8%) in California and 1.7% (95% CI, 1.0%–2.8%) in DC. All other state-specific estimates for these products were unstable (relative standard error >30%) or had zero respondents report current use of the corresponding tobacco product.

c Women who reported ever use of at least 2 of the 6 tobacco products assessed, and reported using the respective products “every day” or “some days” at the time of the survey. For cigarettes only, users were defined as women who had smoked ≥100 cigarettes during their lifetime and now smoked cigarettes either “every day” or “some days.”

d Women who reported having smoked ≥100 cigarettes during their lifetime and smoked “every day” or “some days” at the time of survey administration.

e Women who reported having used the respective product at least once during their lifetime and used “every day” or “some days” at the time of the survey.

f Unstable estimates not presented because of relative standard error >30%.

The adjusted likelihood of any current tobacco product use, current cigarette smoking, and current e-cigarette use among women significantly varied by all examined sociodemographic factors (Table 2). The likelihood of any current tobacco product use was highest among women aged 35 to 44 years (aPR, 1.87; 95% CI, 1.67–2.09) compared with those aged 18 to 24 years, and lowest among Hispanic women (aPR, 0.29; 95% CI, 0.25–0.32) compared with non-Hispanic White women. The likelihood of any current cigarette smoking was highest among women aged 35 to 44 years (aPR, 2.69; 95% CI, 2.33–3.11) compared with women aged 18 to 24 years, and lowest among Hispanics (aPR, 0.26; 95% CI, 0.23–0.30) compared with non-Hispanic Whites as well as women with a college degree or higher (aPR, 0.26; 95% CI, 0.23–0.29) compared with those with less than a high school education. The likelihood of any current e-cigarette use was highest among women who were never married/divorced/widowed (aPR, 1.69; 95% CI, 1.39–2.05) and lowest among women aged ≥65 years compared with those aged 18 to 24 years (aPR, 0.13; 95% CI, 0.10–0.18).

**Table 2:** Adjusted Prevalence Ratios of Any Current Tobacco Product Use, Cigarette Smoking, and E-cigarette Use^a^ Among Women, by Selected Demographics, Tobacco Use Supplement to the Current Population Survey, United States, 2018–2019

Characteristic	Current Any Tobacco Product Use		Current Cigarette Smoking		Current E-Cigarette Use	
	Weighted % (95% CI)	aPR^b^ (95% CI)	Weighted % (95% CI)	aPR^b^ (95% CI)	Weighted % (95% CI)	aPR^b^ (95% CI)
**Overall**	11.6 (11.3–11.9)	—	10.0 (9.7–10.3)	—	1.8 (1.7–1.9)	—
**Age, y**						
18–24	10.0 (9.0–11.2)	1 [Reference]	6.1 (5.3–7.1)	1 [Reference]	3.9 (3.3–4.5)	1 [Reference]
25–34	12.9 (12.2–13.5)	1.76 (1.58–1.97)	10.3 (9.6–10.9)	2.32 (2.01–2.69)	2.6 (2.2–2.9)	0.96 (0.79–1.17)
35–44	12.5 (11.9–13.2)	1.87 (1.67–2.09)	11.0 (10.4–11.6)	2.69 (2.33–3.11)	1.8 (1.5–2.0)	0.74 (0.60–0.92)
45–64	14.3 (13.8–14.8)	1.68 (1.49–1.89)	13.2 (12.7–13.6)	2.50 (2.16–2.90)	1.6 (1.4–1.8)	0.50 (0.40–0.63)
≥65	6.9 (6.5–7.3)	0.53 (0.47–0.60)	6.3 (5.9–6.7)	0.76 (0.65–0.90)	0.6 (0.5–0.8)	0.13 (0.10–0.18)
**Race/ethnicity^c^**						
White	13.5 (13.1–13.9)	1 [Reference]	11.8 (11.4–12.2)	1 [Reference]	2.3 (2.2–2.5)	1 [Reference]
American Indian/Alaska Native	20.9 (17.5–24.8)	1.09 (0.90–1.32)	18.1 (14.6–22.2)	1.07 (0.86–1.32)	3.7 (2.2–6.2)	1.04 (0.58–1.85)
Asian/Pacific Islander	3.5 (2.9–4.3)	0.34 (0.27–0.41)	2.8 (2.2–3.5)	0.31 (0.25–0.39)	0.6 (0.4–0.9)	0.31 (0.19–0.50)
Black	12.7 (11.9–13.6)	0.62 (0.57–0.66)	10.5 (9.8–11.3)	0.58 (0.53–0.63)	0.7 (0.5–1.0)	0.20 (0.15–0.27)
Hispanic	5.5 (5.0–6.2)	0.29 (0.25–0.32)	4.5 (4.0–5.1)	0.26 (0.23–0.30)	0.8 (0.6–1.1)	0.24 (0.18–0.33)
Other	20.8 (17.7–24.3)	1.23 (1.05–1.45)	16.8 (13.9–20.2)	1.18 (0.98–1.43)	4.6 (2.9–7.1)	1.23 (0.77–1.96)
**Education**						
<High school	16.5 (15.3–17.7)	1 [Reference]	14.9 (13.8–16.1)	1 [Reference]	1.7 (1.3–2.2)	1 [Reference]
High school	16.0 (15.4–16.6)	0.85 (0.78–0.92)	14.2 (13.7–14.8)	0.82 (0.76–0.89)	2.4 (2.2–2.7)	1.23 (0.91–1.66)
Some college	13.8 (13.3–14.3)	0.70 (0.65–0.76)	11.6 (11.2–12.2)	0.66 (0.60–0.71)	2.5 (2.2–2.7)	1.13 (0.83–1.53)
College degree or more	5.2 (4.9–5.6)	0.30 (0.27–0.33)	4.1 (3.7–4.3)	0.26 (0.23–0.29)	0.9 (0.7–1.0)	0.43 (0.31–0.60)
**Annual household income, $**						
<25,000	17.7 (16.9–18.5)	1 [Reference]	15.7 (15.0–16.5)	1 [Reference]	2.3 (2.0–2.6)	1 [Reference]
25,000–49,999	13.2 (12.6–13.9)	0.85 (0.80–0.91)	11.7 (11.1–12.3)	0.85 (0.79–0.91)	2.0 (1.7–2.3)	0.94 (0.76–1.15)
50,000–99,999	10.9 (10.4–11.4)	0.76 (0.71–0.81)	9.1 (8.7–9.6)	0.72 (0.70–0.78)	1.9 (1.7–2.2)	0.93 (0.75–1.14)
≥100,000	6.0 (5.5–6.4)	0.51 (0.46–0.57)	4.6 (4.3–5.0)	0.46 (0.41–0.52)	1.2 (1.0–1.4)	0.67 (0.52–0.86)
**Marital status**						
Married/ living with partner	8.6 (8.2–8.9)	1 [Reference]	7.5 (7.2–7.8)	1 [Reference]	1.2 (1.1–1.4)	1 [Reference]
Never married	13.5 (12.9–14.2)	1.44 (1.34–1.53)	10.5 (9.9–11.1)	1.40 (1.30–1.50)	3.0 (2.7–3.4)	1.69 (1.39–2.05)
Divorced/widowed/ separated	15.3 (14.8–15.9)	1.55 (1.45–1.65)	14.1 (13.6–14.6)	1.54 (1.44–1.65)	1.7 (1.6–2.0)	1.61 (1.34–1.95)
**Disability status^d^**						
No	10.6 (10.2–10.9)	1 [Reference]	8.9 (8.7–9.2)	1 [Reference]	1.7 (1.6–1.9)	1 [Reference]
Yes	18.9 (18.1–19.9)	1.44 (1.36–1.54)	17.2 (16.3–18.1)	1.42 (1.33–1.52)	2.4 (2.1–2.8)	1.63 (1.34–1.98)
**US region^e^**						
Northeast	11.3 (10.6–12.0)	1 [Reference]	9.7 (9.0–10.3)	1 [Reference]	1.7 (1.5–2.1)	1 [Reference]
Midwest	14.6 (13.8–15.4)	1.10 (1.01–1.19)	12.8 (12.1–13.5)	1.12 (1.03–1.21)	2.2 (1.9–2.6)	1.03 (0.83–1.27)
South	12.1 (11.6–12.6)	1.00 (0.93–1.07)	10.3 (9.9–10.8)	1.00 (0.93–1.07)	1.8 (1.6–2.0)	1.04 (0.85–1.27)
West	8.6 (8.1–9.1)	0.86 (0.80–0.93)	7.3 (6.8–7.7)	0.86 (0.79–0.94)	1.5 (1.3–1.8)	0.89 (0.70–1.13)

Abbreviations: aPR, adjusted prevalence rate; e-cigarettes, electronic cigarettes.

a Current any tobacco product use includes women who reported ever use of at least 1 of the 6 tobacco products assessed (cigarettes, e-cigarettes, cigars, water pipe or hookah, smokeless tobacco [moist snuff, dip, spit, chew tobacco, or snus], and regular pipe), and reported using the respective product “every day” or “some days” at the time of the survey. For current any tobacco product use, a total of n=188 female respondents with missing current tobacco use status across all products were excluded. For cigarettes, current smokers were defined as women who had smoked ≥100 cigarettes during their lifetime and now smoked cigarettes either every day or some days. For e-cigarettes, current users were defined as women who ever used e-cigarettes and reported using the respective product “every day” or “some days.”

b Model-adjusted for age, race, education, annual household income, marital status, and US region.

c Whites, American Indians/Native Americans, Asians/Pacific Islanders, Blacks and others are non-Hispanic; Hispanic women could be of any race.

d Includes any of the following: deaf or difficulty of hearing; blind or has serious difficulty seeing even when wearing glasses; serious difficulty concentrating, remembering, or making decisions because of a physical, mental, or emotional condition; serious difficulty walking or climbing stairs; difficulty doing errands alone such as visiting a doctor’s office or shopping because of a physical, mental, or emotional condition.

e Northeast: Connecticut, Maine, Massachusetts, New Hampshire, New Jersey, New York, Pennsylvania, Rhode Island, and Vermont. Midwest: Illinois, Indiana, Iowa, Kansas, Michigan, Minnesota, Missouri, Nebraska, North Dakota, Ohio, South Dakota, and Wisconsin. South: Alabama, Arkansas, Delaware, District of Columbia, Florida, Georgia, Kentucky, Louisiana, Maryland, Mississippi, North Carolina, Oklahoma, South Carolina, Tennessee, Texas, Virginia, and West Virginia. West: Alaska, Arizona, California, Colorado, Hawaii, Idaho, Montana, Nevada, New Mexico, Oregon, Utah, Washington, and Wyoming.

## Discussion

This study underscores the heterogeneity of tobacco use patterns among women across multiple tobacco products. Based on state-specific estimates, during 2018–2019, at least 1 in 15 women currently used some form of tobacco product. Multiple social, environmental, and personal factors influence tobacco use among women (1). Consistent with prior research (1,2,6), prevalence was highest among American Indian/Alaska Native women and non-Hispanic women of other races, women with a disability, and those with low annual household income and educational attainment. Moreover, although cigarette smoking among women has declined over time (7), tobacco product use among women across states is still primarily driven by combustible tobacco product use from cigarette smoking.

In alignment with 2019 national findings (8), e-cigarettes were the second most commonly used tobacco product among women. Though our study demonstrates that prevalence of current multiple tobacco product use among women was low overall, others’ examination of the 2018–2019 TUS-CPS data indicate that approximately 7.5% of female current smokers reported current e-cigarette use nationally (9). E-cigarettes have the potential to benefit adult nonpregnant smokers if used as a complete substitute for those unable to quit regular cigarettes or other combustible tobacco products (10). However, evidence to conclude that e-cigarettes, in general, increase smoking cessation is inadequate (10). As the tobacco product landscape continues to evolve, public health messaging efforts can emphasize to women that all tobacco products carry inherent risks and that multiple tobacco product users are at increased risk for nicotine addiction and dependence (2,11).

Our study has limitations. First, tobacco product use among women was self-reported, and corresponding estimates may be subject to response bias or underreporting. Second, small state-specific sample sizes for the use of certain tobacco products led to imprecise estimates that could not be presented. Third, pregnancy status, which could further affect tobacco product use estimates among women of reproductive age, was not identified. Finally, these estimates may differ from those of other national and state-based surveys, due in part to varying survey methods, fielding timelines, and definitions of current use.

Taken together, these data can inform targeted interventions among subgroups of women with higher rates of tobacco product use, as well as the regulation of tobacco products in coordination with comprehensive, evidence-based, population-based strategies (2,9,12) to prevent and reduce the burden of tobacco product use among all women.
